# Genomic insights into the fungal lignocellulolytic system of *Myceliophthora thermophila*

**DOI:** 10.3389/fmicb.2014.00281

**Published:** 2014-06-18

**Authors:** Anthi Karnaouri, Evangelos Topakas, Io Antonopoulou, Paul Christakopoulos

**Affiliations:** ^1^Biotechnology Laboratory, Department of Synthesis and Development of Industrial Processes, School of Chemical Engineering, National Technical University of AthensAthens, Greece; ^2^Biochemical Process Engineering, Chemical Engineering, Department of Civil, Environmental and Natural Resources Engineering, Luleå University of TechnologyLuleå, Sweden

**Keywords:** *Myceliophthora thermophila*, plant biomass, lignocellulolytic enzymes, CAZy, biofuels

## Abstract

The microbial conversion of solid cellulosic biomass to liquid biofuels may provide a renewable energy source for transportation fuels. Cellulolytic fungi represent a promising group of organisms, as they have evolved complex systems for adaptation to their natural habitat. The filamentous fungus *Myceliophthora thermophila* constitutes an exceptionally powerful cellulolytic microorganism that synthesizes a complete set of enzymes necessary for the breakdown of plant cell wall. The genome of this fungus has been recently sequenced and annotated, allowing systematic examination and identification of enzymes required for the degradation of lignocellulosic biomass. The genomic analysis revealed the existence of an expanded enzymatic repertoire including numerous cellulases, hemicellulases, and enzymes with auxiliary activities, covering the most of the recognized CAZy families. Most of them were predicted to possess a secretion signal and undergo through post-translational glycosylation modifications. These data offer a better understanding of activities embedded in fungal lignocellulose decomposition mechanisms and suggest that *M. thermophila* could be made usable as an industrial production host for cellulolytic and hemicellulolytic enzymes.

## Introduction

Ethanol production from lignocellulosic biomass, comprised primarily of cellulose and hemicellulose, appears to evolve as one of the most important technologies for sustainable development. Given its renewable nature, biomass is a potential raw material not only for the production of biofuels, but also chemicals, energy and other materials of main industrial interest (Zhang et al., 2006). The monosaccharides contained in the cellulosic (glucose) and hemicellulosic fractions (xylose, arabinose, mannose, and galactose) represent substrates that can be used for ethanol production via fermentation. To initiate the degradation of these fractions, it is necessary to overcome the physical and chemical barriers presented by the cohesive combination of the main biomass components, which hinders the hydrolysis of cellulose and hemicellulose into fermentable sugars. The above include high substrate viscosity, poor mass transfer conditions and long reaction times, during which hydrolysis reactors are susceptible to contamination. Fungi are the main decomposers of lignocellulosic biomass in terrestrial ecosystems and the enzymes they secrete to break down lignocellulose may be useful in industrial processes. Thermophilic fungi provide a potential source of plant cell wall degrading enzymes with higher levels of specific activity and better stability at higher temperatures, thus making it feasible to minimize the hydrolysis time, reduce substrate viscosity and contamination levels (Margaritis and Merchant, 1986).

*Myceliophthora thermophila* (synonym *Sporotrichum thermophile*) is a thermophilic filamentous fungus, classified as an ascomycete, which was isolated from soil in eastern Russia and constitutes an exceptionally powerful cellulolytic organism, which synthesizes a complete set of enzymes necessary for the breakdown of cellulose. The growth rate and cell density of this microorganism appear to be similar in media containing cellulose or glucose (Bhat and Maheshwari, 1987). The 38.7 Mbp genome of *M. thermophila*, comprising about 9500 genes, organized in 7 chromosomes, has been sequenced and annotated (Joint Genome Institute, University of California, http://genome.jgi-psf.org; Berka et al., 2011). It revealed a large number of genes putatively encoding industrially important enzymes, such as carbohydrate-active enzymes (CAZy), proteases, oxido-reductases, and lipases, while more than 200 sequences have been identified exclusively for plant cell-wall-degrading enzymes. These sequences encode a large number of glycoside hydrolases (GH) and polysaccharide lyases, covering the most of the recognized families (Table 1). In addition, *M. thermophila* was developed into a proprietary mature enzyme production system with easy scaling (C1 strain; Visser et al., 2011). The main features of C1 are the high production levels (up to 100 g/L protein), as well as the maintenance of low viscosity levels of the culture medium, thus enabling fermentation process to reach very high densities.

**Table 1:** **Number of predicted CAZymes encoded in the genome of *M. thermophila***.

**Specific activity**		**CAZy module(s)**	**No. id. seq**.
Cellulases	Endoglucanases	GH 5, 7, 12, 45	8
	Cellobiohydrolases	GH 6, 7	7
	β-glucosidases	GH 1, 3	8
	LPMOs	AA9	25
Xylanases	Xylanases	GH 10, 11	12
	Xylosidases	GH 3, 43	4
Arabinases	Endoarabinases	GH 43	3
	Exo-arabinases/ arabinofuranosidases	GH 43, 51, 62	11
Mannanases	Endomannanases	GH 5, 26	3
	Mannosidases	GH 2	2
Pectinases	Polygalacturonases	GH28	2
	Rhamnosidases	GH78	1
	Pectin lyases	PL 1, 3, 4, 20	8
	Pectin esterases	CE 8, 12	4
Esterases	Feruloyl esterases	CE 1	4
	Acetyl esterases	CE 3, 5, 16	8
	Acetylmannan esterases	CE 12	2
	Glycuronoyl esterases	CE 15	2

GHs, Glycoside hydrolases; CEs, carbohydrate esterases; and PLs, polysaccharide lyases are included, covering the most of the recognized families.

*M. thermophila* exhibits an impressing number of accessory enzymes belonging to AA9 (previously described as GH61) and family 1 carbohydrate binding modules (CBM), which are the highest found in fungi (Berka et al., 2011). Family 1 CBM presents a cellulose-binding function and is almost exclusively found in enzymes of fungal origin (http://www.cazy.org; Guillén et al., 2009). In addition, *M. thermophila* distinguishes itself from other cellulolytic fungi, such as *Aspergillus niger* and *Trichoderma reesei* by the presence of a relatively high number of (glucurono) arabinoxylan degrading enzymes (Hinz et al., 2009). Eleven putative xylanases were found that belong into GH 10 and 11 families compared to five in both *A. niger* (Broad Institute of Harvard and MIT, http://www.broadinstitute.org) and *T. reesei* (Joint Genome Institute, University of California, http://genome.jgi-psf.org), while 14 arabinofuranosidases belonging to GH 43, 51, and 62 families were found compared to 13 in *A. niger* and three in *T. reesei*, rendering *M. thermophila* a promising source of hemicellulolytic enzymes. Studying the secretome of *M. thermophila* after 30 h of growth in barley and alfalfa straws, it was found to comprise of 683 predicted proteins, 230 of which are proteins with unknown function (Berka et al., 2011). Based on transcriptome analysis, many secreted enzymes including accessory enzymes, hypothetical proteins and proteins with unknown function were upregulated, when the fungus is grown in more complex substrates, such as agricultural straws, compared to glucose, indicating their crucial role in lignocellulose degradation (Berka et al., 2011).

*M. thermophila* grows in temperatures between 25 and 55°C, while a relative growth performance study on mycobroth agar plates indicated that the optimum condition is at 45°C (Morgenstern et al., 2012). The temperature optima for several enzymes with the same specific activity, characterized from *M. thermophila*, range from 50 to 70°C. For example, *StEG5* endoglucanase, expressed in *A. niger*, exhibits a T_opt_ of 70°C (Tambor et al., 2012), while recombinant *MtEG7* expressed in *Pichia pastoris* exhibited an optimal temperature of 60°C (Karnaouri et al., 2014). The same characteristic is also observed for *M. thermophila* xylanases expressed in *A. niger*, showing optimal activity at temperatures between 50 and 70°C (Berka et al., 2011), underpinning the enzymatic potential that is not only diverse in catalytic activities, but also in properties increasing its efficiency in various temperatures.

Individual cellulolytic enzymes exhibit comparable activities on cellulose; however, synthetically composed multienzyme mixtures display a much higher performance than those from other lignocellulolytic thermostable fungi (Szijártó et al., 2011; Zhang et al., 2013). This can be attributed to synergistic mode of action between the enzymes. For example, synergism between GH 11 xylanase and type C feruloyl esterase has been proved (Moukouli et al., 2010), as well as between cellobiohydrolases acting on the reducing and the non-reducing end of cellulose molecules (Gusakov et al., 2007).

In this review, an overview will be given of the cellulolytic and hemicellulolytic potential of *M. thermophila* regarding the degradation of plant cell wall material. The genomic potential of this thermophilic fungus demonstrates a strong enzymatic toolbox including hydrolytic, oxidative and accessory activities that may enhance its ability to decompose plant biomass. Many of these enzymes have been isolated from culture supernatant or selectively overexpressed in *M. thermophila* (C1 strain) or in other heterologous hosts and have been characterized. All sequences used in this study were extracted from Genome Portal database (http://genome.jgi-psf.org) and the continually updated CAZy database (http://www.cazy.org/; Lombard et al., 2014). The conserved domains were found with Pfam/InterProscan (http://pfam.sanger.ac.uk/; Punta et al., 2012), while the theoretical molecular mass and isoelectric point for each protein were calculated using the ProtParam tool of ExPASY (http://web.expasy.org/protparam/). Post-translational glycosylation sites were predicted with NetNGlyc 1.0 server (http://www.cbs.dtu.dk/services/NetNGlyc/) and NetOGlyc 3.1 server (http://www.cbs.dtu.dk/services/NetOGlyc/). Predicted secretome was extracted using SignalP v4.0 (http://www.cbs.dtu.dk/services/SignalP/).

## Cellulolytic system

Cellulose is composed of β-D-anhydroglucopyranose units linked by (1,4)-glycosidic bonds. Polymorphism or allotropy refers to the existence of more than one crystalline forms differing in physical and chemical properties. Cellulose degradation is attributed to the synergistic action of three complementary enzyme activities: (1) endoglucanases (EGs, EC 3.2.1.4); (2) exoglucanases, including cellodextrinases (EC 3.2.1.74) and cellobiohydrolases (CBHs, EC 3.2.1.91 for the non-reducing end acting CBHs and EC 3.2.1.176 for the reducing end acting ones) and (3) β-glucosidases (BGs, EC 3.2.1.21) (Lynd et al., 2002). Amorphous regions of the polysaccharide chain are cleaved randomly by EGs, while CBHs remove processively cellooligosaccharides from chain ends. The latter are the most abundant enzymes in the secretome of cellulolytic fungi (Jun et al., 2011; Ribeiro et al., 2012). Their main representatives are GH family 7 (CBH I) that attack the reducing end of a cellulose chain and GH family 6 (CBH II) that are specific toward the non-reducing end of the chain. Until very recently, CBHs were considered as the main degraders of the crystalline part of cellulose (Sweeney and Xu, 2012).

EGs are widespread among GH families, with examples described for families 5–9, 12, 44, 45, 48, 51, and 74 on the continually updated CAZy database (http://www.cazy.org/; Lombard et al., 2014). Most of them show optimal activity at neutral or acidic pH and at temperatures below 50°C (Maheshwari et al., 2000). Exo-glucanases (or CBHs) act in a processive manner (Davies and Henrissat, 1995) and are classified only to two families, as referred previously. One of the important features of all CBHs is that they can act on microcrystalline cellulose (Terri, 1997). BGs include enzymes of GH1 and GH3 families that hydrolyze cellobiose and short (soluble) cellooligosaccharides to glucose that could subsequently fermented to ethanol; e.g., the hydrolysis reaction is performed in the liquid phase, rather than on the surface of the insoluble cellulose particles, such as EGs and CBHs. The removal of cellobiose is an important step of the enzymatic hydrolysis process, as it assists in reduction of the inhibitory effect of cellobiose on EG and CBH. BG activity has often been found to be rate-limiting during enzymatic hydrolysis of cellulose (Duff and Murray, 1996; Tolan and Foody, 1999), and due to that the commercial cellulase enzyme preparations are often supplemented with BG activity.

Until recently, only hydrolytic enzymes were thought to play a role in the degradation of recalcitrant cellulose and hemicelluloses to fermentable sugars. Recent studies demonstrate that enzymes from the GH family 61 show lytic polysaccharide monooxygenase activity (LPMO) and have an enhancing cellulolytic effect when combined with common cellulases (Horn et al., 2012). Together with cellobiose dehydrogenase (CDH; EC 1.1.99.18), an enzymatic system capable of oxidative cellulose cleavage is formed, which increases the efficiency of cellulases and boosts the enzymatic conversion of lignocellulose. It has long been thought that the proteins of GH family 61 are accessory proteins enhancing cellulose decomposition. They were thus frequently referred to as the “cellulose enhancing factors” (Harris et al., 2010) and previously thought to have no or only weak endoglucanase activity (Karlsson et al., 2001). Now, these enzymes are now reclassified to AA9 family of CAZy database and their mode of action provide a new dimension to the classical concept of cellulose degradation, as recently reviewed by Dimarogona et al. (2013). These copper-dependent enzymes were shown to cleave cellulose by an oxidative mechanism provided that reduction equivalents from CDH or low molecular weight reducing agents (e.g., ascorbate) are available (Langston et al., 2011). In some genomes, AA9 genes even outnumber cellulose genes. It remains to be elucidated whether all of these encoded enzymes have PMO activity, but their large number emphasizes the importance of oxidative cellulose cleavage. *M. thermophila'*s genome has 25 AA9 genes, encoding putative proteins acting as accessory LPMOs enzymes (Berka et al., 2011). This number is outstanding in comparison to common lignocellulolytic organisms, as *A. niger* (seven sequences) and *T. reesei* (nine sequences). This difference can explain the high efficiency of hydrolysis of *Myceliopthora* in nature substrates and reveals the crucial role of these enzymes in the whole procedure.

**Figure 1:**
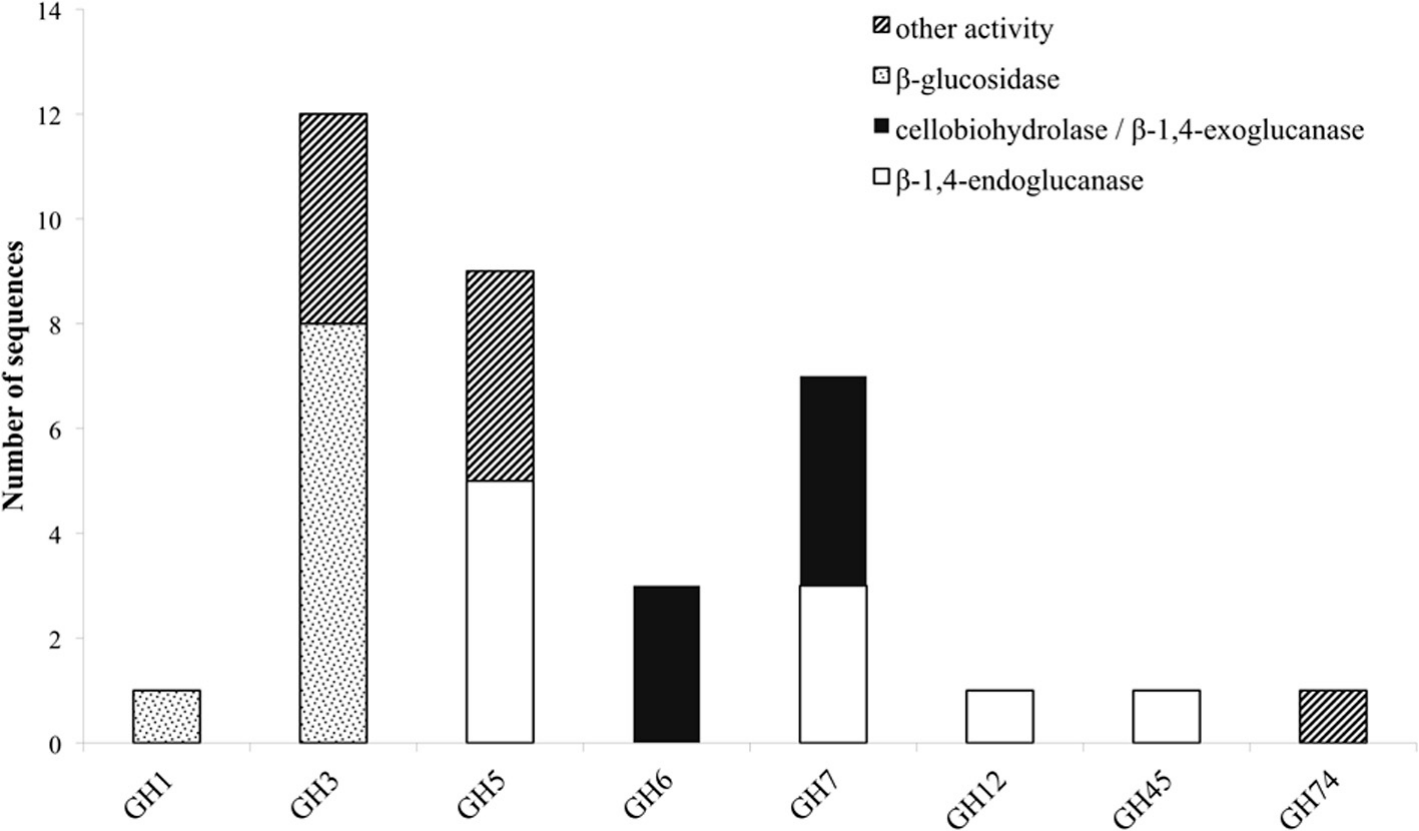
**Distribution of cellulolytic enzymes of *M. thermophila* throughout eight GH families**. Other activities refer to β-xylosidase (GH3), β-1,6-galactanase, β-1,3-glucanase, endo-1,4-beta-mannosidase or putative proteins with unknown function (GH5). GH74 represents xyloglucan specific 1,4-endoglucanase/xyloglucanase.

Throughout the genome of *M. thermophila*, there are eight sequences encoding EG activity, seven sequences of CBH activity and nine sequences of BG activity (Figure 1; Table 2). The theoretical average molecular weight of the translated proteins is calculated at 51.05 ± 16.2 kDa and the theoretical pI at 5.58 ± 0.3. EGs are distributed to families GH5, 7, 12, and 45, all predicted to possess a secretion signal and several *N*- and *O*-glycosylation sites. Only two of them exhibit a CBM that belong in family 1. CBHs represent three non-reducing acting enzymes of GH6 family and four reducing-end acting enzymes of GH7 family. All of these enzymes seem to be targeted to secretion pathway and modified with glycans during post-translational modifications. BGs are classified to GH3 family, except one GH1 sequence, while none of them exhibit a CBM, as expected. Four are secreted and have potential *N*- and *O*-glycosylation sites, showing the highest molecular weight compared to the other cellulases, with a theoretical average value of 85.18 ± 3.2 kDa.

**Table 2:** **Number of predicted sequences encoding enzymes with cellulolytic activity (EGs, CBHs, and BGLs)**.

**Protein_ ID**	**InterPro_ ID**	**InterPro_ description**	**CAZy module**	**GenBank ID**	**Location**	**Secretion signal**	**Exons**	**pI/MW (kDa)**	**Length**	**N-gly positions**	**O-gly positions**	**CBM1**	**BLAST parameters**
**EGs**													
MYCTH_ 52068	IPR017853	Glycoside hydrolase, catalytic core	GH5	AEO58455.1	4:188041–189524	18aa	7	4.66/35.2	319aa	3	2	−	
MYCTH_ 86753	IPR017853	Glycoside hydrolase, catalytic core	GH5	AEO53769.1	1:2823610–2825549	16aa	3	5.07/40.85	389aa	3	17	+	65% identity with endoglucanase GH5 from *Thermoascus aurantiacus* [PDB ID: 1GZJ] and 79% with β-1,4-endoglucanase from *Penicillium brasilianum* [GenBank: ACB06750]
MYCTH_ 94336	IPR017853	Glycoside hydrolase, catalytic core	GH5	AEO57401.1	3:908597–909880	21aa	1	4.92/44.6	406aa	2	4	−	61% identity with cellulase family protein from *Ophiostoma piceae* UAMH 11346 [GenBank: EPE03278.1]
MYCTH_ 43356	IPR017853	Glycoside hydrolase, catalytic core	GH5	AEO53175.1	1:580471–581733	26aa	1	7.86/44.18	394aa	1	2	−	71% identity with EG from endoglucanase *Verticillium dahliae* [GenBank: EGY21718.1]
MYCTH_ 116157	IPR017853	Glycoside hydrolase, catalytic core	GH7	AEO59361.1	4:4081701-4083768	20aa	2	4.75/47.20	436aa	2	2	−	68% identity with endo-1,4-beta-glucanase from *Humicola grisea* [UniProtKB/Swiss-Prot:Q12622.1]
MYCTH_ 111372	IPR017853	Glycoside hydrolase, catalytic core	GH7	AEO58196.1	3:4135959–4137568	22aa	1	4.61/46.632	442aa	2	16	+	64% identity with endoglucanase I from *Hypocrea orientalis* [GenBank: AFD50194.1]
MYCTH_ 109444	IPR008985	Concanavalin A-like lectin/glucanases superfamily	GH12	AEO60532.1	6:212893–214302	15aa	3	5.46/25.48	247aa	−	−	−	56% identity with Cel12A from *Humicola grisea* [PDB ID: 1OLR] and 63% with endoglucanase from *Fusarium oxysporum* [GenBank: ENH72093.1]
MYCTH_ 76901	IPR014733	Barwin-like endoglucanase	GH45	AEO54078.1	1:4001048–4002222	18aa	4	4.66/21.8	207aa	−	3	−	78% identity with endoglucanase from *Humicola insolens* [PDB ID: 1HD5] and 82% with endoglucanase from *Humicola grisea* [GenBank: BAA74957.1]
**CBHs**													
MYCTH_ 51545	IPR016288	1,4-beta cellobiohydrolase	GH6	AEO59280.1	4:3726723–3728113	17aa	3	4.66/40.64	378aa	−	4	−	49% identity with CBHII from *Trichoderma reesei* [GenBank: ADC83999.1] and 51% with CBHII from *Trichoderma reesei* [PDB ID: 3CBH]
MYCTH_ 66729	IPR016288	1,4-beta cellobiohydrolase	GH6	AEO55787.1	2:46305–48489	17aa	4	5.28/49.41	465aa	1	35	+	79% identity with CBH II from *Humicola insolens* [PDB ID: 1BVW] and 64% with CBHII from *Trichoderma viride* [GenBank: AAQ76094.1]
MYCTH_ 2303045	IPR016288	1,4-beta cellobiohydrolase	GH6	AEO57190.1	2:5389544–5391132	18aa	2	5.94/39.41	363aa	4	5	−	56% identity with endoglucanase Cel6b from *Humicola insolens* [PDB ID: 1DYS] and 39% with Cellobiohydrolase II (Cel6a) from *Humicola insolens* [1BVW]
MYCTH_ 109566	IPR008985	Concanavalin A-like lectin/glucanases superfamily	GH7	AEO55544.1	1:9753507–9755507	17aa	2	4.77/54	509aa	1	20	+	61% identity with CBH I from *Humicola grisea* [GenBank: BAA09785.1] and 67% with cellobiohydrolase Cel7d (Cbh 58) from *Phanerochaete chrysosporium* [PDB: 1GPI]
MYCTH_ 42937	IPR008985	Concanavalin A-like lectin/glucanases superfamily	GH7	AEO53522.1	1:1922814–1924693	20aa	5	4.34/47.08	436aa	2	2	−	66% identity with β-1,4-cellobiohydrolase from *Phanerochaete chrysosporium* [UniProtKB: P13860.1]
MYCTH_ 97137	IPR008985	Concanavalin A-like lectin/glucanases superfamily	GH7	AEO61262.1	7:24661–26448	20aa	6	4.48/46.5	430aa			−	79% identity with β-1,4-beta-cellobiosidase from *Melanocarpus albomyces* [GenBank: CAD56667.1]
MYCTH_ 95095 fragment	IPR008985	Concanavalin A-like lectin/glucanases superfamily	GH7	AEO58824.1	4:1967106–1967560	19aa	2	6.87/11.82	109aa	1	4	C-term, no prediction	77% identity with β-1,4-beta-cellobiosidase from *Acremonium thermophilum* [GenBank: CAM98446.1]
**BGLs**													
MYCTH_ 115968	IPR017853	Glycoside hydrolase, catalytic core	GH1	AEO57459.1	3:1092854–1095039	−	2	5.53/54.1	476aa	1	−	−	92% identity with β-glucosidase from *Humicola grisea* [GenBank: BAA74958.1]
MYCTH_ 38200	IPR017853	Glycoside hydrolase, catalytic core	GH3	AEO61246.1	6:4096305–4099311	−	2	5.54/105.3	968aa	7	3	−	78% identity with β-glucosidase from *Chaetomium thermophilum* var. *thermophilum* [GenBank: EGS22574.1]
MYCTH_ 2059579	IPR017853/ IPR026891	Glycoside hydrolase, catalytic core/fibronectin type III-like domain	GH3	AEO56238.1	2:1843191–1846185	23aa	4	5.94/94.8	880aa	6	5	−	57% identity with β-1,4-glucosidase from *Marssonina brunnea* [GenBank: EKD11918.1]
MYCTH_ 66804	IPR017853/ IPR026891	Glycoside hydrolase, catalytic core/fibronectin type III-like domain	GH3	AEO58343.1	3:4861135–4863642	17aa	2	4.99/75.83	716aa	2	2	−	72% identity with β-glucosidase from *Trichoderma reesei* [PRF: 227874]
MYCTH_ 80304	IPR017853/ IPR026891	Glycoside hydrolase, catalytic core/fibronectin type III-like domain	GH3	AEO58175.1	3:3949561–3952737	19aa	4	5.05/93.3	851aa	11	−	−	77% identity with β-glucosidase from *Chaetomium thermophilum* [GenBank: ABR57325.2]
MYCTH_ 62925	IPR017853	Glycoside hydrolase, catalytic core	GH3	AEO53892.1	1:3330354–3333832	−	7	5.39/97.05	884aa	3	4	−	76% identity with β-glucosidase from *Fusarium oxysporum* [GenBank: EMT71863.1]
MYCTH_ 2302509	IPR026891	Fibronectin type III-like domain	GH3	AEO56946.1	2:4612453–4614052	−	1	5.15/47.75	440aa	6	2		60% identity with β-glucosidase from *Grosmannia clavigera* [GenBank: EFX01027.1]
MYCTH_ 58882	IPR017853/ IPR026891	Glycoside hydrolase, catalytic core/fibronectin type III-like domain	GH3	AEO60477.1	6:51326–53769	16aa	2	5.21/82.46	761aa	5	−		
MYCTH_ 2129052 (fragment)	IPR001764	Glycoside hydrolase, family 3, N terminal	GH3	AEO59952.1	5:2414101–2414414	−	2	4.70/92.48	83aa	1	−		46% identity with β-glucosidase from *Parastagonospora avenae* [GenBank: CAB82861.1]

Most of them predicted to possess a secretion signal and several N- and O-glycosylation sites, thus undergoing through post-translational modifications.

Totally, 12 cellulases have been isolated and characterized (Table 3). The group of Bukhtojarov et al. (2004) investigated the properties of individual cellulases from the multienzyme complex produced by a mutant strain of *M. thermophila* C1 (Visser et al., 2011). Among EGs, the highest saccharification activity was displayed by EG60 and EG51, representing enzymes of 60 and 51 kDa, respectively, which exhibited pI values of 3.6 and 5.0, respectively. It has been shown later that the EG51 and EG60 represent the GH5 and GH7 EGs from *M. thermophila*, respectively, (Gusakov et al., 2011). A different EG (*St*Cel5A) displays a typical GH5 domain, exhibiting optimal activity at pH 6.0 and 70°C and retained greater than 50% of its activity following 2 h of incubation at 55°C, diluted in 10 mM citrate buffer pH 4.5 (Tambor et al., 2012). A GH7 EG gene was functionally expressed in methylotrophic yeast *P. pastoris* and subsequently characterized (Karnaouri et al., 2014). Substrate specificity analysis revealed that the enzyme is one of the most thermostable fungal enzymes reported up to now and exhibits high activity on substrates containing β-1,4-glycosidic bonds as well as activity on xylan-containing substrates. Moreover, *Mt*EG7a was proved to liquefy rapidly and efficiently pretreated wheat straw, indicating EGs' key role to the initial step of hydrolysis of high-solids lignocellulose substrates (Karnaouri et al., 2014). This change in viscosity of these substrates is probably due to the gradual reduction of the average chain length of cellulose polysaccharides by endo-acting enzymes, such as endoglucanases. Totally, four CBHs and two BGs have been isolated from *M. thermophila* crude supernatant and studied. CBH IIb is the product of MYCTH_66729 gene that represents an enzyme of GH6 family, which is attached to polysaccharide substrate through a CBM and exhibits high levels of activity in comparison to other CBHs (Gusakov et al., 2007). In the same study, the isolation of CBH Ib, a GH7 family enzyme (MYCTH_2140736) is reported, which acts mainly against microcrystalline cellulose and CMC. Bukhtojarov et al. (2004) studied the properties of CBH Ia and CBH IIa, which are classified to GH7 and GH6 family, respectively. CBH Ia is the product of MYCTH_109566 gene, and seems to be expressed in two isoforms with distinct molecular weights, one exhibiting the catalytic domain owing a CMB and the other only the catalytic domain and part of the linker, after proteolysis. This enzyme is produced as a major protein of fungi's secretome (20–25% of the total extracellular protein) and adsorbed strongly on microcrystalline cellulose. It has been shown that there is a significant synergism between CBH IIb and CBH Ia enzymes during substrate hydrolysis (Gusakov et al., 2007).

**Table 3:** **Description of the characterized cellulolytic enzymes either isolated from the culture broth of a *M. thermophila* C1 mutant strain or expressed in a heterologous host**.

**Enzyme**	**Type of action**	**GH**	**MW-monomer (kDa)**	**pH_opt_**	**T_opt_ (°C)**	**pI**	**Gene**	**Source**	**References**
StCel5A	Endoglucanase	5	46	6	70	ND		cDNA library-EST analysis, expessed in *A. niger*	Tambor et al., 2012
EG51	Endoglucanase	5	51	4.7	70	4.8		Isolated from the culture broth of a C1 mutant strain	Bukhtojarov et al., 2004; Gusakov et al., 2005
MtEG7a	Endoglucanase	7	65	5	60	Multiple bands in 3.8–4.5	*eg7a*	Expressed in *P. pastoris*	Karnaouri et al., 2014
EG60	Endoglucanase	7	60	4.7	60	3.7		Isolated from the culture broth of a C1 mutant strain	Bukhtojarov et al., 2004
EG28	Endoglucanase	12	28	5.35	60	5.7		Isolated from the culture broth of a C1 mutant strain	Bukhtojarov et al., 2004
EG25	Endoglucanase	45	25	5.5	65	4		Isolated from the culture broth of a C1 mutant strain	Bukhtojarov et al., 2004; Gusakov et al., 2005
CBH Ia	1,4-beta cellobiohydrolase	7	65	5	ND	4.5		Isolated from the culture broth of a C1 mutant strain	Bukhtojarov et al., 2004; Gusakov et al., 2005
CBH IIa	1,4-beta cellobiohydrolase	6	43	5.4	65	4.2		Isolated from the culture broth of a C1 mutant strain	Bukhtojarov et al., 2004; Gusakov et al., 2005
CBH Ib	1,4-beta cellobiohydrolase	7	60	ND	ND	ND		Isolated from the culture broth of a C1 mutant strain	Gusakov et al., 2007
CBH IIb	1,4-beta cellobiohydrolase	6	70	ND	ND	5.6		Isolated from the culture broth of a C1 mutant strain	Gusakov et al., 2007
MtBgl3a	beta-glucosidase	3	90	5	70	4.0	*bgl3a*	Expressed in *P. pastoris*	Karnaouri et al., 2013
Bxl5	beta-glucosidase	3	120 ± 5	4.6	75	5.2	*bxl5*	Homologously expressed in C1	Dotsenko et al., 2012

Most of them exhibit optimum temperatures above 60°C and pH around 5.0.

Recently, another type of specific activity was revealed. Xyloglucan specific exo-β-1,4-glucanase (Xgl74A; EC 3.2.1.155) is classified to GH74 family and catalyzes the hydrolysis of (1-4)-D-glucosidic linkages in xyloglucans aiming in the successful removal of oligosaccharides from the chain end (Grishutin et al., 2004). Xyloglucan is a major structural polysaccharide found in the primary cell walls of higher plants that interact with cellulose microfibrils via hydrogen bonds to form a structural network that is assumed to play a key role in cell wall integrity. It consists of a cellulose-like backbone of β-1,4-linked D-glucopyranose (D-Glcp) residues, which most of them are substituted at C-6 with α-d-Xylp-(1→6) residues, to which other saccharides may be attached (most frequently, d-Galp and l-Fucp). *M. thermophila* was found to produce an exo-β-1,4-glucanase (Xgl74A) (Grishutin et al., 2004) with high specific activity toward tamarind xyloglucan, and very low or absent activity against carboxymethylcellulose (CMC) and barley β-glucan. Due to its unique substrate specificity the enzyme was given a new number in the Enzyme Nomenclature (EC 3.2.1.155). Apart from Xgl74A, tw*o* out of the seven cellulases reported from *M. thermophila* (Cel12A and Cel45A) possess a notable activity against xyloglucan, together with their major activities toward CMC and barley β-glucan (Bukhtojarov et al., 2004).

## Hemicellulolytic system

**Figure 2:**
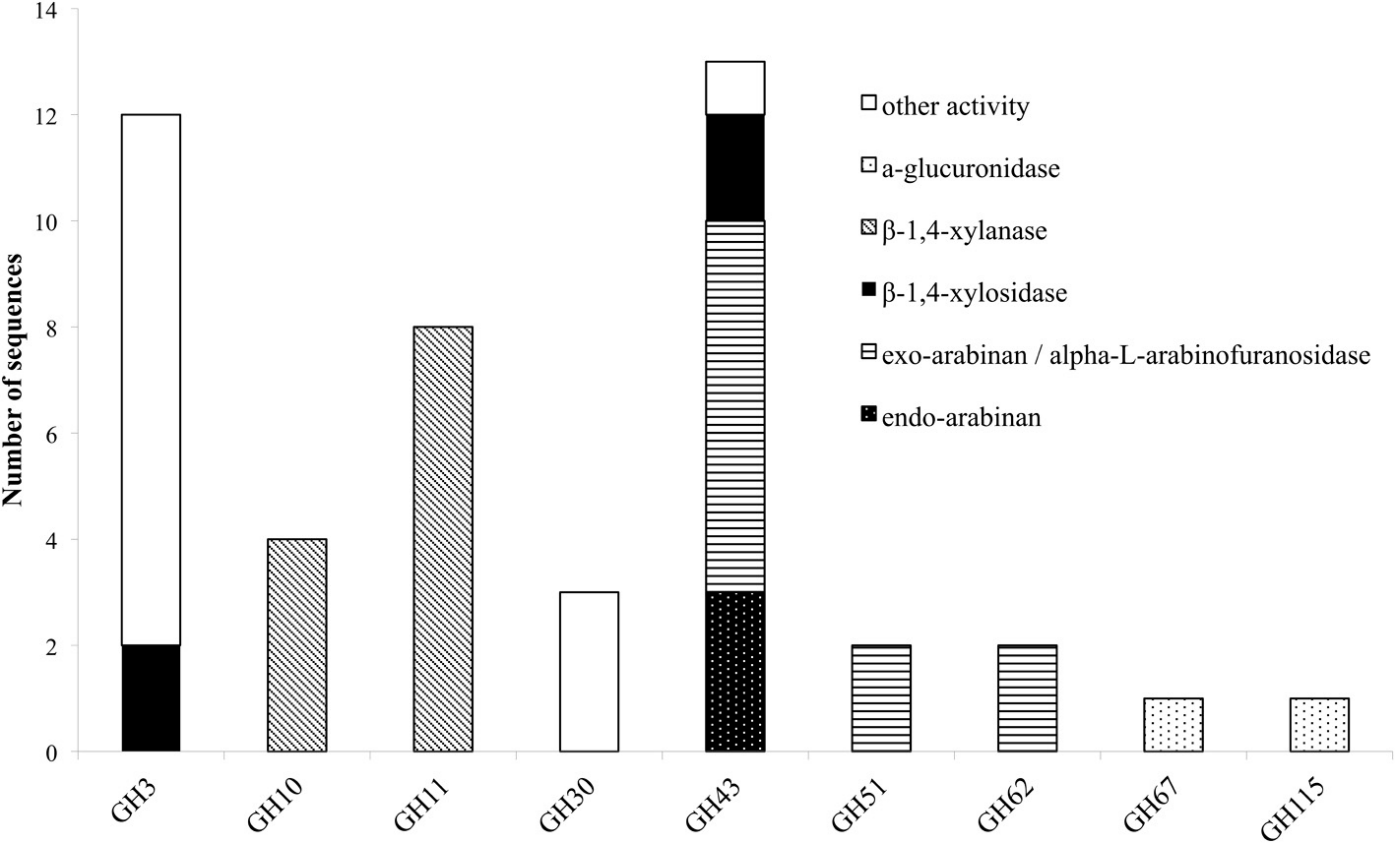
**Distribution of hemicellulolytic enzymes of *M. thermophila* throughout nine GH families**. Other activities refer to β-glycosidase (GH3), xylanase with endo-exo mode of action and xylobiohyrolase (GH30), and galactan 1,3-beta-galactosidase (GH43).

**Figure 3:**
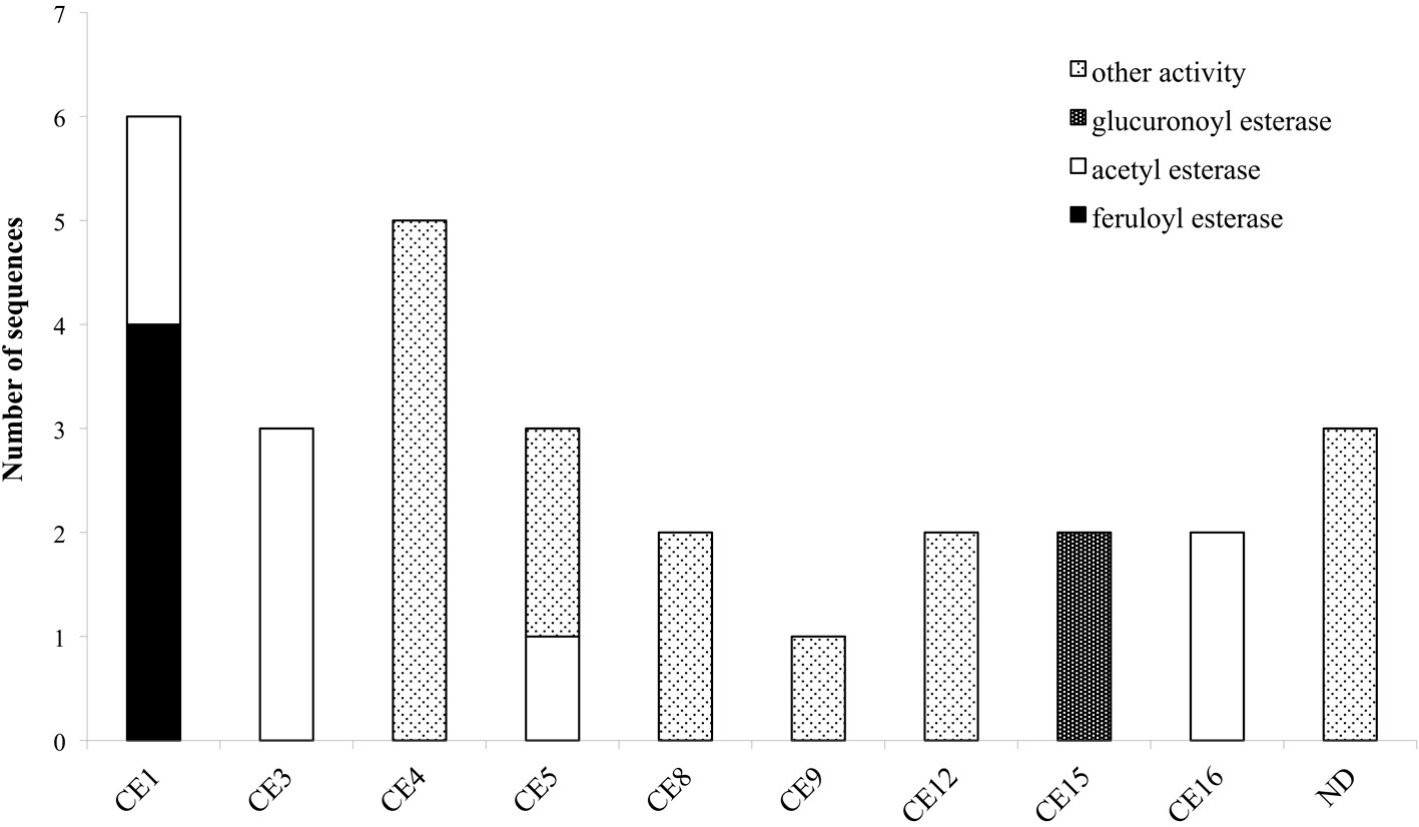
**Distribution of hemicellulolytic enzymes of *M. thermophila* throughout nine CE families**. Family CE4 is comprised of putative proteins with polysaccharide deacetylase activity, CE5 of cutinases and CE8, 12 of pectin esterases. ND (not determined) refers to sequences encoding putative proteins with unknown activity which are not classified to a specific family.

Hemicellulose polymers have a much more diverse structure than cellulose and consequently several enzymes are needed to completely degrade the polysaccharide into monosaccharides. Xylan that is the major component of hemicellulose in the plant cell wall, is consisted of a β-D-(1,4)-linked xylopyranosyl backbone, which, depending on the origin, can be substituted with arabinofuranosyl, 4-0-methylglucopyranosyl, feruloyl and acetyl groups (Shibuya and Iwasaki, 1985). Feruloyl groups can form strong networks through peroxidase-catalyzed oxidative coupling forming diferuloyl bridges (Topakas et al., 2007). The main enzymes needed for depolymerization are xylanases, assisted by accessory enzymes such as β-xylosidases and different arabinofuranosidases making the xylan backbone more accessible (Sørgensen et al., 2007). Other accessible enzymes that enhance xylan degradation are acetyl-xylanesterases (Poutanen et al., 1990), ferulic acid esterases (Topakas et al., 2007), and α-glucuronidases (De Vries et al., 1998). *M. thermophila'*s hemicellulase genes are organized in 10 GH families (3, 10, 11, 30, 43, 51, 62, and 67) (Figure 2) and nine carbohydrate esterase (CE) families (1, 3, 4, 5, 8, 9, 12, 15, and 16) (Figure 3). Many of the encoding proteins have been isolated from the WT culture supernatant or expressed in heterologous hosts and finally characterized in terms of specific activity and physicochemical properties. The majority of them are predicted to follow the secretion pathway, while modified with *N*- and/or *O*- glucans, comprising a total amount of 66 enzymes that act synergistically for the degradation of hemicellulose.

### Xylanases/xylosidases

The degradation of xylan requires the concerted action of a number of powerful enzymes with varying specific activities, including xylanases and β-xylosidases. Xylanases (endo-1,4-β-xylanases, EC 3.2.1.8) are enzymes hydrolyzing β-1,4-glycosidic linkages in the backbone of xylans, while most of them belong to GH family 10 or 11 based on amino acid similarities and structural features (Henrissat, 1991). GH10 xylanases exhibit less substrate specificity than GH11 enzymes and can hydrolyze different types of decorated xylans, while GH11 xylanases are highly specific and do not tolerate many decorations on the xylan backbone (Biely et al., 1997). β-Xylosidases (EC 3.2.1.37) hydrolyze the soluble xylo-oligosaccharides and xylobiose from the non-reducing end liberating xylose, produced by the activity of xylanases. These enzymes play an important role in xylan degradation by relieving the end product inhibition of endoxylanases (Knob et al., 2010). The genome of *M. thermophila* encodes totally 12 xylanases with endo- mode of activity, classified to GH 10 and 11 and four xylosidases, classified to GH3 and 43 families (Table 4). All aforementioned xylan-degrading translated sequences, apart from three, are predicted to exhibit a potential secretion signal. Xylanases possess 1-3 *N*-glycosylation and several *O*-glycosylation sites, whereas more *N*-sites are predicted for xylosidases, though not all of them are glycosylated during post-translational modifications. GH family 30 contains two genes encoding xylanolytic enzymes with endo-exo activity and one sequence for a characterized xylobiohydrolase, releasing xylobiose units from the substrate (Emalfarb et al., 2012).

**Table 4:** **Number of predicted sequences encoding enzymes with hemicellulolytic activity (β-1,4-xylanases and β-xylosidases)**.

**Protein_ ID**	**InterPro_ ID**	**InterPro_ description**	**CAZy module**	**GenBank ID**	**Location**	**Secretion signal**	**Exons**	**pI/MW (kDa)**	**Length**	**N-gly positions**	**O-gly positions**	**Conserved domains**	**BLAST parameters**
**XYLs**													
MYCTH_ 2139438	IPR013781	Glycoside hydrolase, catalytic domain	GH10	AEO58598.1	4:852357–853828	19aa	3	6.42/39.65	356aa	1	−		84% identity with XynA from *Humicola insolens* [GenBank: AGG68962.1]
MYCTH_ 52904	IPR013781	Glycoside hydrolase, catalytic domain	GH10	AEO59320.1	4:3932271–3933431	16aa	3	5.70/34.20	311aa	1	1		77% identity with endo-1,4-beta-xylanase protein from *Chaetomium thermophilum* [GenBank: EGS20178.1]
MYCTH_ 2125938 (fragment)	IPR013781	Glycoside hydrolase, catalytic domain	GH10	AEO56947.1	2:4614086–4614433	ND	1	10.11/ 13.53	115aa	1	1		63% identity with XynA from *Humicola insolens* [GenBank: AGG68962.1]
MYCTH_ 112050	IPR013781	Glycoside hydrolase, catalytic domain	GH10	AEO60457.1	5:4306110–4307709	17aa	2	5.52/ 42.86	396aa	−	22	CBM1	66% identity with endo-1,4-beta-xylanase A from *Gaeumannomyces graminis* [GenBank: EJT78185.1]
MYCTH_ 100068	IPR008985	Concanavalin A-like lectin/glucanases superfamily	GH11	AEO57157.1	2:5265193–5266157	19aa	2	7.77/ 27.53	259aa	1	1	CBM1	74% identity with endo-beta1,4-xylanase from *Chaetomium gracile* [GenBank: BAA08650.1]
MYCTH_ 89603	IPR008985	Concanavalin A-like lectin/glucanases superfamily	GH11	AEO55365.1	1:9130476–9131260	20aa	2	6.71/ 23.12	208aa	1	1		87% identity with endo-beta-1,4-xylanase from *Chaetomium* sp. CQ31 [GenBank: ADW78258.1]
MYCTH_ 2121801	IPR008985	Concanavalin A-like lectin/glucanases superfamily	GH11	AEO62054.1	7:3382576–3383357	18aa	2	7.81/22.59	200aa	1	2		86% identity with endo-1,4-beta-xylanase protein from *Chaetomium thermophilum* [GenBank: EGS23409.1]
MYCTH_ 99786	IPR008985	Concanavalin A-like lectin/glucanases superfamily	GH11	AEO54512.1	1:5821400–5822224	21aa	2	5.50/ 22.17	205aa	1	2		56% identity from xylanase II from *Hypocrea orientalis* [GenBank: AFD50199.1]
MYCTH_ 2309574 (fragment)	IPR008985	Concanavalin A-like lectin/glucanases superfamily	GH11	AEO60385.1	5:4039395–4039724	ND	1	9.19/ 7.10	66aa	1	−		63% identity with xyn11C from *Chaetomium thermophilum* [GenBank: CAD48751.1]
MYCTH_ 2309575 (fragment)	IPR008985	Concanavalin A-like lectin/glucanases superfamily	GH11	AEO60386.1	5:4039732–4040297	−	1	5.49/ 16.34	146aa	2	4		77% identity with endoxylanase 11C from *Chaetomium thermophilum* [GenBank: CAD48751.1]
MYCTH_ 56237	IPR008985	Concanavalin A-like lectin/glucanases superfamily	GH11	AEO59539.1	5:215065–215872	18aa	2	4.83/ 22.08	205aa	1	2		72% identity with xylanase from *Scytalidium thermophilum* [GenBank: BAD07040.1]
MYCTH_ 49824	IPR008985	Concanavalin A-like lectin/glucanases superfamily	GH11	AEO58284.1	3:4545212–4546113	16aa	2	5.93/ 23.79	214aa	3	1		79% identity with endo-1,4-beta-xylanase from *Chaetomium cupreum* [GenBank: ABI48363.1]
**BXYLs**													
MYCTH_ 104628	IPR017853	Glycoside hydrolase, catalytic core	GH3	AEO60531.1	6:209038–211675	−	2	4.69/89.69	835aa	6	1	BglX	55% identity with 1,4-beta-xylosidase from *Fusarium fujikuroi* IMI 58289 [GenBank: CCT61496.1]
MYCTH_ 50705	IPR017853/ IPR026891	Glycoside hydrolase, catalytic core/fibronectin type III-like domain	GH3	AEO58346.1	3:4871063–4873395	21aa	2	5.57/79.51	739aa	6	2		53% identity with 1,4-beta-xylosidase from *Neurospora crassa* [GenBank: CAB91343.2]
MYCTH_ 80104	IPR008985	Concanavalin A-like lectin/glucanases superfamily	GH43	AEO58399.1	3:5027172–5028785	−	1	5.86/ 61.23	537aa	1	−	XynB	80% identity with beta-xylosidase from *Fusarium oxysporum* [GenBank: EMT73385.1]
MYCTH_ 2072383	IPR008985	Concanavalin A-like lectin/glucanases superfamily	GH43	AEO61672.1	7:1863035–1864780	19aa	2	4.77/53.52	494aa	4	3	XynB	45% identity with beta-xylosidase from *Micromonospora* sp. ATCC39149 [NCBI Ref: WP_ 007073468.1]

β-1,4-xylanases are classified to GH10 and GH11 families, while β-xylosidases to GH43 family. Most of them predicted to possess a secretion signal and several N- and O-glycosylation sites.

Ten xylanases have been purified and characterized from multienzyme preparations of *M. thermophila* modified strains (Ustinov et al., 2008; van Gool et al., 2013). Four of them, belonging to GH10 family (Table 5), are the products of two genes, either with the presence of a family 1 CBM or displaying only the catalytic domain after partial proteolytic digestion (Ustinov et al., 2008). These enzymes, thought classified to the same family, can hydrolyze different types of decorated xylans. They differ in degradation of high and low substituted substrates and the substitution pattern seems to be an important factor influencing their efficiency (van Gool et al., 2012). Six xylanases, belonging to GH11 family, represent true xylanases, with high specific activities against glucuronoxylans and arabinoxylans. Four of these enzymes exhibit lower thermostability in comparison to GH10 xylanases, in which extended glycosylation has been noticed (Ustinov et al., 2008). One showed a substrate specificity pattern similar to GH10 enzymes and secreted in two forms, with or without CBM (van Gool et al., 2013).

**Table 5:** **Description of the characterized β-1,4-xylanases isolated from the culture broth of a *M. thermophila* C1 mutant strain**.

**Enzyme**	**CAZy module**	**MW-monomer (kDa)**	**pH_opt_**	**T_opt_ (°C)**	**pI**	**Gene**	**References**
Xyn10A	GH10	42/31^*^	5.5–7.0	65–70	7.8/8.9	*xyl1*	Ustinov et al., 2008; van Gool et al., 2012
Xyn10B	GH10	57/46^*^	5.5–7.0	80–85	4.4/4.3	*xyl3*	Ustinov et al., 2008; van Gool et al., 2012
Xyn10C	GH11	40	5.0	80	4.8	*xyl4*	Ustinov et al., 2008
Xyn11A	GH11	24	6.5	70	7.9	*xyl2*	Ustinov et al., 2008
Xyn11B	GH11	23	6.0–6.5	65–70	8.4	*xyl6*	Ustinov et al., 2008
Xyn11C	GH11	22	4.5	65	6.7	*xyl5*	Ustinov et al., 2008
Xyl7	GH11	22/30^*^	5.5–6.5	50–60	7.3/7.6	*xyl7*	van Gool et al., 2013
Xyl8	GH11	22	5.5–6.0	50–65	6.2	*xyl8*	van Gool et al., 2013

They display optimal activity at temperatures between 50 and 70 °C, increasing fungi's hydrolytic efficiency in various temperatures. Marked proteins

(^*^) were isolated in two different forms, with (high molecular weight enzyme) or without CBM (low molecular weight enzyme).

### Arabinohydrolases

L-arabinose is widely present in various hemicellulosic biomass components, such as arabinoxylan, where the main β-D-(1,4)-linked xylopyranosyl backbone is substituted with arabinose residues. α-L-arabinofuranosidases (AFase; EC 3.2.1.55) are enzymes that release arabinofuranose residues substituted at position *O*-2 or *O*-3 of mono or di-substituted xylose residues (Gruppen et al., 1993). Apart from that, AFases act in synergism with other arabinohydrolases, endo-(1,5)-α-L-arabinanases (ABNase; EC 3.2.1.99) for the decomposition of arabinan, a major pectin polysaccharide. Arabinan consists of a backbone of α-(1,5)-linked L-arabinofuranosyl residues, some of which are substituted with α-(1,2)- or α-(1,3)-linked arabinofuranosides (Weinstein and Albersheim, 1979). Degradation of arabinan polymer to arabinose sugars is driven by the synergistic action of two major enzymes, AFases and ABNases (Kim, 2008). AFases specifically catalyze the hydrolysis of terminal non-reducing L-arabinofuranosyl residues from arabinan, while the resulting debranched backbone could be efficiently hydrolyzed by endo-acting ABNases, thus generating a variety of arabino-oligosaccharides with an inverting mode of action (Beldman et al., 1997). Thoughout CAZy families, arabinohydrolases belong to the GH family 43, 51, 54, 62, and 93 (Figure 3).

The genome of the *M. thermophila* encodes 14 enzymes that putatively release arabinose or arabinose oligomers from arabinan (Hinz et al., 2009). Eleven sequences contain a secretion signal peptide and produced as extracellular or cell-bounded proteins, while almost all of them exhibit isoelectric point around 4.6–5.6 (Table 6). Seven of them have been selectively overexpressed homologously in *M. thermophila* C1 host and found to release arabinose from wheat arabinoxylan polymers and oligomers (Hinz et al., 2009). *M. thermophila* arabinofuranosidases are selective in releasing arabinose from either single or double substituted xylose residues in arabinoxylans. Eight enzymes, belonging to GH families 43, 51, 62, and 93 with different type of arabinolytic activity have been purified and characterized (Hinz et al., 2009; Kühnel et al., 2011; Pouvreau et al., 2011b) (Table 7).

**Table 6:** **Number of predicted sequences encoding enzymes with hemicellulolytic activity (endoarabinases and arabinofuranosidases)**.

**Protein_ID**	**InterPro_ID**	**InterPro_description**	**CAZy module**	**GenBank ID**	**Location**	**Secretion signal**	**Exons**	**pI/MW (kDa)**	**Length**	**N-gly positions**	**O-gly positions**	**Conserved domains**	**BLAST parameters**
MYCTH_39555	IPR006710	Glycoside hydrolase family 43	GH43	AEO61077.1	6:3204367–3205350	−	1	4.87/37.12	327aa	−	1		82% identity with a-N-arabinofuranosidase/alpha-L-arabinofuranosidase from *Fusarium fujikuroi* [GenBank: CCT69715.1]
MYCTH_2303298	IPR006710	Glycoside hydrolase family 43	GH43	AEO57303.1	3:514704–516772	23aa	1	4.91/59.16	535aa	1	1	XynB	55% identity with arabinase from *Auricularia delicata* [GenBank: EJD41599.1]
MYCTH_2305738	IPR006710	Glycoside hydrolase family 43	GH43	AEO58423.1	4:27829–29545	19aa	4	4.90/33.18	301aa	1	2		52% identity with a-N-arabinofuranosidase 2 from *Fusarium oxysporum* [GenBank: EMT68952.1]
MYCTH_103032	IPR016840	Glycoside hydrolase family 43, endo-1,5-alpha-L-arabinosidase	GH43	AEO58422.1	4:25889–27048	20aa	3	5.63/32.74	301aa	1	4		60% identity with arabinan endo-1,5-alpha-L-arabinosidase A from *Aspergillus kawachii* [GenBank: GAA90221.1]
MYCTH_2300677	IPR006710	Glycoside hydrolase family 43	GH43	AEO56123.1	2:1449574–1451195	22aa	1	4.79/41.72	410aa	5	35		38% identity with endo-arabinase from *Colletotrichum gloeosporioides* [GenBank: ELA34066.1]
MYCTH_2064169	IPR006710	Glycoside hydrolase family 43	GH43	AEO58916.1	4:2252824–2255361	16aa	11	6.42/64.17	573aa	7	5	XynB	65% identity with arabinofuranosidase from *Coprinopsis cinerea* [NCBI Ref: XP_001836293.2] and 52% with beta-xylosidase from *Aspergillus oryzae* [GenBank: EIT77121.1]
MYCTH_2301869	IPR006710	Glycoside hydrolase family 43	GH43	AEO56692.1	2:3685597–3687704	18aa	1	4.89/65.71	613αα	3	1	Xyl1	55% identity with Xylosidase/arabinosidase (arabinofuranoside) from *Fusarium oxysporum* [GenBank: ENH67211.1]
MYCTH_2127683	IPR007934	a-L-arabinofuranosidase B	GH43	AEO58631.1	4:1109767–1110916	−	3	4.91/36.35	339aa	1	3	CBM42	65% with a-N-arabinofuranosidase from *Chaetomium* sp. [GenBank: AFU88757.1]
MYCTH_2306666	IPR023296	Glycosyl hydrolase, five-bladed-beta-propellor domain	GH43	AEO58919.1	4:2260967–2262690	25aa	4	5.26/35.76	329aa	1	1		72% identity with arabinosidase (arabinofuranosidase) from *Glomerella graminicola* [GenBank: EFQ32395.1]
MYCTH_42071	IPR010720	a-L-arabinofuranosidase, C-terminal	GH51	AEO53569.1	1:2109152–2111615	18aa	6	5.80/69.54	636aa	6	2		54% identity with a-L-arabinofuranosidase from *Penicillium chrysogenum* [GenBank: BAH70480.1]
MYCTH_83019	IPR010720	a-L-arabinofuranosidase, C-terminal	GH51	AEO58452.1	4:178906–181205	−	5	5.81/57.67	512aa	2	2		75% identity with a-L-arabinofuranosidase from *Glomerella graminicola* [GenBank: EFQ27215.1]
MYCTH_98003	IPR005193	Glycoside hydrolase, family 62, arabinosidase	GH62	AEO60934.1	6:2448019–2449251	22aa	2	5.52/37.84	353aa	−	3	CBM1	73% identity with a-L-arabinofuranosidase axhA-2 from *Colletotrichum higginsianum* [GenBank: CCF42219.1]
MYCTH_55982	IPR005193	Glycoside hydrolase, family 62, arabinosidase	GH62	AEO59813.1	5:1711636–1712601	19aa	1	4.90/33.1	302aa	1	4		77% identity with a-L-arabinofuranosidase from *Streptomyces ghanaensis* [NCBI Ref: WP_004979944.1]
MYCTH_104827	IPR011040	Sialidases	GH93	AEO55492.1	1:9532077–9534361	17aa	2	5.16/41.8	378aa	3			56% with exo-arabinanase from *Penicillium chrysogenum* [NCBI Ref: XP_002562032.1]

All of them are selectively produced in culture supernatant and contain a secretion signal peptide, being produced as extracellular or cell-bounded proteins.

**Table 7:** **Description of the characterized endoarabinases and arabinofuranosidases isolated from the culture broth of a *M. thermophila* C1 mutant strain**.

**Enzyme**	**CAZy module**	**Mode of action**	**MW-monomer (kDa)**	**pH_opt_**	**T_opt_(°C)**	**Uniprot/SwissProt ID**	**Gene**	**Source**	**References**
Abf1	GH62	Arabinofuranosidase/releases O-2 or O-3 arabinose from mono-substituted xylose					*abf1*	Selectively produced in C1-host	Hinz et al., 2009
Abf2	GH62	Arabinofuranosidase/releases O-2 or O-3 linked arabinofuranosyl residues from mono-substituted xylose					*abf2*	Selectively produced in C1-host	Hinz et al., 2009
Abf3	GH51	Arabinofuranosidase/releases arabinose from the non-reducing end of reduced arabinose oligomers	70	5	40	HQ324254	*abf3*	Selectively produced in C1-host	Pouvreau et al., 2011b
Abn7	GH43	Arabinofuranosidase/releases O-3 linked arabinofuranosyl residues from di-substituted xylose	70	5	40	HQ324255	*abn7*	Selectively produced in C1-host	Pouvreau et al., 2011b
Abn1	GH43	Endoarabinase	36	5.5	60	HQ324251	*abn1*	Overexpressed in fermentation supernatant	Kühnel et al., 2011
Abn2	GH93	Exoarabinase/arabinobiose from the non-reducing end of reduced arabinose oligomers	40	4	50		*abn2*	Overexpressed in fermentation supernatant	Kühnel et al., 2011
Abn4	GH43	Arabinofuranosidase/releases arabinose from the non-reducing end of reduced arabinose oligomers	33	5.5	60	HQ324253	*abn4*	Overexpressed in fermentation supernatant	Kühnel et al., 2011
Abn5		Arabinofuranosidase					*abn5*	Selectively produced in C1-host	Hinz et al., 2009

Abn7 and Abf3 are GH43 and GH51 arabinases respectively, which were selectively produced in C1 host. Abn7 was found to hydrolyze arabinofuranosyl residues at position O-3 of double substituted xylosyl residues in arabinoxylan-derived oligosaccharides, while Abf3 released arabinose from position *O*-2 or *O*-3 of single substituted xyloses. When these enzymes were incubated together, in combination with a GH10 endo-xylanase for the hydrolysis of arabinoxylans, they resulted in a synergistic increase in arabinose release from the substrate (Pouvreau et al., 2011b). In addition, a-L-arabinohydrolases Abn1, Abn2, and Abn4 were overexpressed in C1 and the produced culture supernatant has been shown to produce neutral branched arabino-oligosaccharides from sugar beet arabinan by enzymatic degradation. As found by sugar analysis, neutral arabino-oligosaccharides contained an α-(1,5)-linked backbone of l-arabinosyl residues and carried single substituted α-(1,3)-linked l-arabinosyl residues or consisted of a double substituted α-(1,2,3,5)-linked arabinan structure within the molecule (Westphal et al., 2010). Enzyme Abn4 belongs to GH43 family and is more active toward branched polymeric arabinan substrate that releases arabinose monomers from single substituted arabinose residues, while Abn1 and Abn2 are active toward linear arabinan (Kühnel et al., 2010). Abn2 is a member of GH93 family that consists of exoarabinases acting on linear arabinan, hydrolyzing the α-1,5-linkages of arabinan polysaccharides presented as side chains of pectin. Their mode of action was studied with Abn2, which binds two arabinose units at the subsites −1 and −2 and releases arabinose. Three more arabinohydrolases were also overexpressed in C1 strain (Hinz et al., 2009). Abn5 was found to be specifically active toward arabinan, but not arabinoxylan. Arabinofuranosidases Abf1 and Abf2, members of GH62 family released *O*-2 or *O*-3 substituted arabinose or linked arabinofuranosyl from mono substituted xylose. GH family 62 arabinofuranosidases are reported to be predominantly active toward arabinoxylan and are, therefore, also called arabinoxylan arabinofuranohydrolases (Beldman et al., 1997). Several of these enzymes contain either a CBM1, like Abf1, or a CBM43 (xylan)-binding domain.

### Esterases

The role of esterases in the breakdown of lignocellulosic material is complex and includes the cleavage of bonds between the main hemicellulose part and many types of side chains. So, upon a closer examination of the genome sequences of *M. thermophila*, there is a wide distribution of enzymatic activities through CE families. These enzymes are classified into nine families and their main activities, among others, include the hydrolysis of feruloyl and acetyl ester bonds.

Feruloyl esterases (FAEs; EC 3.1.1.73) are enzymes responsible for cleaving the ester-link between the polysaccharide main chain of xylans and monomeric or dimeric ferulates. They act synergistically with xylanases to release ferulic acid from cell-wall material and can be divided into four groups, namely A–D. The main difference between groups A and D is their substrate specificity toward synthetic substrates and their capability of liberating diferuloyl bridges (Crepin et al., 2004). One of the first FAEs reported from thermophilic fungi, was produced from *M. thermophila* under solid-state fermentation (SSF) conditions. The esterase activity was isolated and partially characterized for its ability to release ferulic acid from complex substrate, destarched wheat bran (Topakas et al., 2003). Two other FAEs, *St*FaeB, a protein with molecular weight of 66 kDa (homodimers of 33 kDa) (Topakas et al., 2004) and *St*FaeC, 46 kDa (homodimers of 23 kDa) (Topakas et al., 2005), were purified to homogeneity from culture supernatants of *M. thermophila*. *St*FaeB hydrolyzed methyl *p*-coumarate, methyl caffeate and methyl ferulate and was active on substrates containing ferulic acid ester linked to the C-5 and C-2 linkages of arabinofuranose. StFaeC showed maximum catalytic efficiency on 4-hydroxy-3-methoxy cinnamate, a substrate with both hydroxyl and methoxy substituents, indicating that it may be the most promising type of FAE as a biocatalyst for the enzymatic feruloylation of aliphatic alcohols, oligo- and polysaccharides. Properties of characterized FAEs are summarized in Table 8. Among the sequences registered to Genome Portal, there are four sequences encoding proteins with catalytic activity of FAE, all belonging to CE family 1. Two of them (MYCTH_48379, MYCTH_39279) seem to be identical with characterized FAEs secreted from *M. thermophila* C1 strain (Kühnel et al., 2012). One sequence (JDI ID: 96478) has been heterologously expressed in *P. pastoris* and encoded a 39 kDa protein (*fae1A*; MtFae1a), which showed high activity toward methyl caffeate and p-coumarate and a strong preference for the hydrolysis of n-butyl and iso-butyl ferulate (Topakas et al., 2012). In addition, MtFae1 esterase release ferulic acid from destarched wheat bran only by the synergistic action of an endo-xylanase (a maximum of 41% total ferulic acid released after 1 h incubation). MYCTH_2302953 sequence has not yet been characterized, however it still shows 66% identity with a type B FAE from *Neurospora crassa* (CAC05587.1). All proteins encoded by the above sequences appear to be secreted and bring several *N*- and *O*-glycosylation sites, as shown in Table 9.

**Table 8:** **Description of *M. thermophila* characterized esterases (FAEs, AcEs, and GEs)**.

**Enzyme**	**Type**	**MW-monomer (kDa)**	**pH_opt_**	**T_opt_ (°C)**	**pI**	**Gene**	**Source**	**References**
**FAEs**								
StFaeB	B	33^*^	6	55–60	3.5		Isolated from culture supernatant	Topakas et al., 2004
StFaeC	C	23^*^	6	55	<3.5		Isolated from culture supernatant	Topakas et al., 2005
FAE	ND	27	8	60	5		Isolated from culture supernatant	Topakas et al., 2003
FaeA1	A	29	6.5	45	≈5.5		Overexpressed in fermentation supernatant	Kühnel et al., 2012
FaeA2	A	36	7.5	40	≈5.2		Overexpressed in fermentation supernatant	Kühnel et al., 2012
FaeB2	B	33	7.5	45	≈6.0		Overexpressed in fermentation supernatant	Kühnel et al., 2012
MtFae1a	B	39	7	50	ND	*fae1a*	Expressed in *P. pastoris*	Topakas et al., 2012
**AcEs**								
MtAxe3	1	33.6	7	40	ND	*axe3*	Overexpressed in fermentation supernatant	Pouvreau et al., 2011a
MtAxe2	5	23.6	7	40	ND	*axe2*	Overexpressed in fermentation supernatant	Pouvreau et al., 2011a
**GEs**								
StGE2	15	43	7	55	ND	*ge2*	Expressed in *P. pastoris*	Topakas et al., 2010
StGE1	15	58	6	60	ND		Isolated from culture supernatant	Vafiadi et al., 2009

* All proteins are monomeric, while in case of FaeB2, dimeric structures are detected, after comparing the results of SDS-PAGE and native electrophoresis.

**Table 9:** **Number of predicted sequences encoding enzymes with hemicellulolytic activity capable of hydrolyzing ester bonds (FAEs, AcEs and GEs)**.

**Protein_ID**	**InterPro_ID**	**InterPro_description**	**CAZy module**	**GenBank ID**	**Location**	**Secretion signal**	**Exons**	**pI/MW (kDa)**	**Length**	**N-gly positions**	**O-gly positions**
**FAEs**											
MYCTH_96478	IPR010126	Esterase, PHB depolymerase	CE1	AEO62008.1	7:3150678–3151912	18aa	3	4.48/29.43	272aa	2	–
MYCTH_48379	SSF53474	Alpha/beta hydrolases	CE1	AEO57132.1	2:5185595–5186503	26aa	1	5.05/29.11	276aa	1	8
MYCTH_39279	SSF53474	Alpha/beta hydrolases	CE1	AEO61094.1	6:3261246–3262256	20aa	2	6.15/27.22	295aa	–	2
MYCTH_2302953	IPR010126	Esterase, PHB depolymerase	CE1	AEO57203.1	2:5187587–5189039	19aa	3	5.82/29.4	276aa	1	–
**AcEs**											
MYCTH_80233	IPR010126	Esterase, PHB depolymerase	CE1	AEO58247.1	3:4383828–4384913	21aa	2	5.93/31.8	294aa	2	–
MYCTH_2130973	SSF53474	Alpha/beta-Hydrolases	CE1	AEO61799.1	7:2287063–2288403	23aa	5	5.03/24.7	233aa	3	–
MYCTH_49700	IPR000675	Cutinase	CE5	AEO58391.1	3:5004208–5005095	17aa	4	5.01/23.4	226aa	1	–
MYCTH_43727	IPR001087	Lipase, GDSL	CE3	AEO54377.1	1:5184409–5185412	18aa	3	4.72/23.2	214aa	–	1
MYCTH_53698	IPR001087	Lipase, GDSL	CE3	AEO59370.1	4:4105354–4106226	18aa	3	5.09/23.7	217aa	1	1
MYCTH_105128	IPR001087	Lipase, GDSL	CE3	AEO60969.1	6:2683598–2685715	21aa	2	9.42/29.6	279aa	–	2
MYCTH_40885	IPR001087/IPR013830	Lipase, GDSL/Esterase, SGNH hydrolase-type	CE16	AEO60664.1	6:1191196–1192286	15aa	2	6.40/38.1	343aa	3	–
MYCTH_84133	IPR001087/IPR013830	Lipase, GDSL/Esterase, SGNH hydrolase-type	CE16	AEO59602.1	5:700951–702131	21aa	2	4.41/32.5	302aa	4	–
**GEs**											
MYCTH_2308381	SSF53474	Alpha/beta-Hydrolases	CE15	AEO60464.1	5:1737542–1739553	18aa	1	5.84/41.7	397aa	–	2
MYCTH_2119719	SSF53474	Alpha/beta-Hydrolases	CE15	AEO60465.1	5:87107–88569	17aa	3	6.44/44.3	417aa	1	1

All of them contain a secretion signal peptide and have a theoretical molecular weight of 28.51 ± 4.1 kDa, ranging between 23 and 39 kDa.

About 60–70% of the xylose residues in hardwood xylan are acetylated at the C2 and/or C3 positions (Lindberg et al., 1973). The complete degradation of acetylated xylans by microbes requires the action of acetyl esterases (AcEs; EC 3.1.1.72), which cleave acetyl side groups from the heteroxylan backbone, and act in synergy with other hemicellulases (Tenkanen et al., 1996). Eight sequences that encode proteins with AcE activity were detected in the genome of *M. thermophila* and showed identity with characterized enzymes. All of them are secreted, as predicted with SignalP and belong to CE families 1, 3, 5, 16 (Table 9). Two of them, Axe2 and Axe3, which bare members of CE5 and CE1 families, respectively, were isolated and characterized (Pouvreau et al., 2011a). Annotated genes, encoding the putative enzymes were cloned into the specially designed *M. thermophila* C1-expression host (Verdoes et al., 2010) and over-produced in the culture medium. Axe2 and Axe3 are able to hydrolyze acetyl groups when they are substituted to the *O*-2 and *O*-3 positions of acetylated xylo-oligosaccharides and complex insoluble polymeric substrates and had a preference for xylooligosaccharides (Pouvreau et al., 2011a).

Glucuronoyl esterases (GEs) are recently discovered enzymes that are suggested to play an important role in the dissociation of lignin from hemicellulose and cellulose by cleaving the ester bonds between the aromatic alcohols of lignin and the carboxyl groups of 4-*O*-methyl-D-glucuronic acid residues in glucuronoxylan (Špániková and Biely, 2006). Sequence alignment studies of these enzymes have revealed a novel conserved amino acid sequence G-C-S-R-X-G that features the characteristic serine residue involved in the mechanism of this esterase family. It has been shown that the mode of action probably involves a nucleophilic serine (Topakas et al., 2010). The genome of *M. thermophila* possesses two genes classified to family CE15 that encode proteins with activity of 4-*O*-methyl-glucuronoyl esterase. Both putative enzymes are secreted and have potential glycosylation sites. The first GE (*St*EG1), isolated from the culture filtrate of *M. thermophila*, was proved to be a thermophilic enzyme that presents a C-terminal CBM, which was active on substrates containing glucuronic acid methyl ester (Vafiadi et al., 2009). Another CE15 protein molecule, *St*GE2 was heterologously expressed in yeast *P. pastoris* and was used to prove that nucleophilic serine residue is responsible for catalytic action of GEs, through site-directed mutagenesis studies (Topakas et al., 2010) and crystal structure determination (Charavgi et al., 2013).

### Mannan-degrading enzymes

Mannan is a great component of hemicellulose, therefore, as expected, the lignocellulolytic toolbox of *M. thermophila* possesses a complete reservoir of genes encoding mannan degrading enzymes. Mannan polymer primarily consists of a backbone structure composed of β-1,4-bound mannose residues or combination of glucose and mannose residues and can be hydrolyzed to its monomers with the synergistic action of β-mannanases (EC 3.2.1.78), β-mannosidases (EC 3.2.1.25), α-galactosidases (EC 3.2.1.22), and acetylmannan esterases (E.C. 3.1.1.6) (McCleary, 1988). The genome of *M. thermophila* encodes three enzymes that putatively catalyze random cleavage of the mannan polysaccharide and belong to GH family 5 and 26. One of these enzymes has been isolated from culture supernatant, characterized and classified as GH5 endo-β-1,4-mannosidase (bMan2, Dotsenko et al., 2012). In addition, there are two genes encoding putative β-mannosidases belonging to GH2 family, while one of them has been characterized in terms of its specificity and physicochemical properties (bMann9, Dotsenko et al., 2012). Two GH27 and one GH26 α-galactosidases boost the efficiency of fungal culture supernatant against hydrolysis of mannan substrate (Emalfarb et al., 2012), while two CE12 family genes encoding proteins with high similarity to known acetyl-mannan esterases have been found.

## Auxiliary enzymes

**Figure 4:**
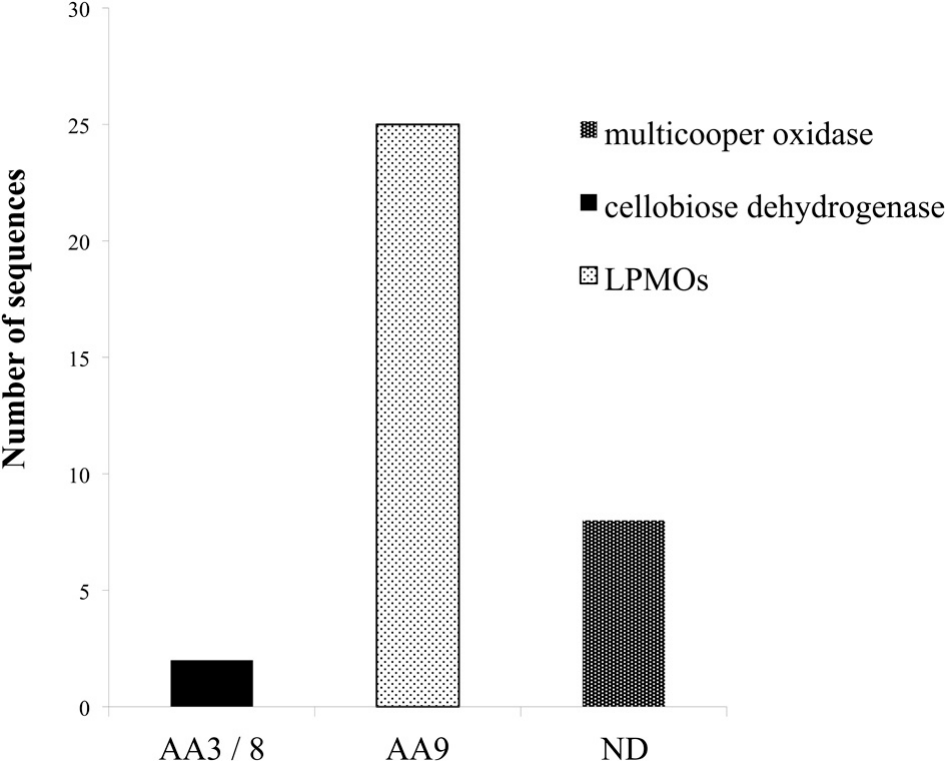
**Distribution of enzymes of *M. thermophila* with auxiliary activities, classified to AA3/8, AA9 families and multicopper oxidases**. *M. thermophila* distinguishes itself from other cellulolytic fungi, exhibiting an impressing number of LPMOs accessory enzymes belonging to AA9 family (previously described as GH61).

In spite of the cooperative activity exhibited by the cellulolytic and hemicellulolytic enzymes, the impressive hydrolytic ability of various microorganisms in nature cannot be attributed only to this endo–exo mechanism. Apart from the hydrolytic system responsible for carbohydrate degradation, it seems that an oxidative system catalyze lignin depolymerization and oxidation of plant cell wall components, yielding reactive molecules (e.g., H_2_O_2_). Recent evidence highlights the critical role of alternative enzymatic partners involved in the oxidation of cell wall components. Among these enzymes, outstanding role during hydrolysis exhibit the originally described as cellulases LPMO enzymes, CDH and multicopper enzymes such as laccases. The genome of *M. thermophila* possesses more than 30 genes that encode proteins with such auxiliary activities (Figure 4). Members of the LPMO family AA9, have been shown to be copper-dependent monooxygenases that enhance cellulose degradation in concert with classical cellulases, as aforementioned before and reviewed by Dimarogona et al. (2013). These enzymes catalyze the cleavage of cellulose by an oxidative mechanism provided that reduction equivalents are available. These equivalents either involve low molecular weight reducing agents (e.g., ascorbate) or are produced by CDH activity (Langston et al., 2011). CDHs are extracellular enzymes produced by various wood-degrading fungi that oxidize soluble cellodextrins, mannodextrins and lactose efficiently to their corresponding lactones by a ping-pong mechanism using a wide spectrum of electron acceptors (Henriksson et al., 2000). Throughout the genome of *M. thermophila*, two genes encoding proteins classified to AA3 and 8 families have been identified (Figure 4). Both of them are predicted to be secreted in the culture supernatant and have potential glycosylation sites. The translated CDH MYCTH_111388 exhibits a C-terminal CBM and a cDNA clone of this sequence has been isolated and biochemically characterized by screening an expression library of *M. thermophila* (Subramaniam et al., 1999). Canevascini et al. (1991) purified a monomeric (91 kDa) and a dimeric (192 kDa) form of CDH that differed not only in molecular weight, but amino acid composition and carbohydrate content. Both forms oxidized cellobiose in the presence of cytochrome c or dichlorophenol–indophenol.

Laccases (EC 1.10.3.1) are multicopper enzymes that catalyze the oxidation of a variety of phenolic compounds, with concomitant reduction of O_2_ to H_2_O. These polyphenol oxidases are produced by most ligninolytic basidiomycetes (Baldrian, 2006) and can degrade lignin and other recalcitrant compounds in the presence of redox mediators (Ruiz-Dueñas and Martínez, 2009). The genome of the *M. thermophila* encodes eight putative enzymes with multicopper oxidase activity. Four of them have been annotated and one (MYCTH_51627) matches the *lcc1* gene product encoding an extracellular laccase (Berka et al., 1997). Four sequences are predicted to possess a secretion signal, while one appears to remain membrane-bound. *Lcc1* gene has been isolated from fungi's genome, heterologously expressed in *A. oryzae* and the produced 85 kDa enzyme (MtL) was characterized as a thermostable low oxidation potential laccase with high reactivity in aqueous medium at room temperature and neutral pH. MtL was tested for its capacity to catalyze enzymatic oxidation of several phenolic and polyphenolic compounds (ferulic acid, gallic acid, caffeic acid, and catechin) (Mustafa et al., 2005). *M. thermophila* laccases have been reported to oxidize lignin surface, by increasing the amount of radicals during thermomechanical pulp fiber material bleaching (Grönqvist et al., 2003) and promote oxidative polymerization of Kraft lignin from back liquor, which is the main by-product of pulp and paper industry (Gouveia et al., 2013).

## Lignocellulosic potential—statistics

**Figure 5:**
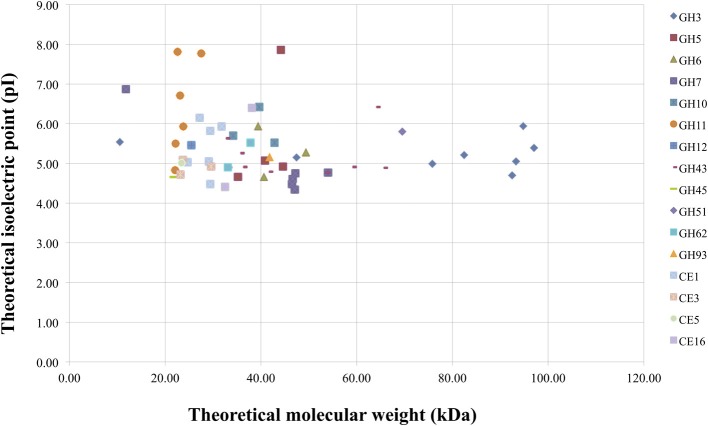
**Theoretical molecular weight of secreted enzymes of *M. thermophila* classified in several GHs and CEs families, plotted against theoretical pI**. The average molecular weight was calculated at 51.05 ± 16.2 kDa (range between 21 and 97 kDa) for cellulolytic enzymes, 35.5 ± 19.5 kDa (range between 22 and 89 kDa) for hemicellulases (GHs/CEs), and 28.51 ± 4.1 kDa (range between 23 and 39 kDa) for the fraction of esterases.

*M. thermophila* is a powerful lignocellulolytic organism, which secretes a complex system of carbohydrate hydrolases for the breakdown of cellulose and hemicellulose, as well as oxidoreductases embedded in lignin degradation. Genome analysis in this review revealed 30 genes encoding cellulases classified to 10 GH families, 66 genes encoding hemicellulases classified to 10 GHs, 9 CEs and 35 genes encoding auxiliary enzymes. The latter include CDHs (AA3/AA8 family), LPMOs (AA9 family) and multicopper oxidases (laccases or laccase-like enzymes). Out of the total consortium of *M. thermophila* sequences encoding proteins with putative lignocellulosic activity, 80.2% are predicted to have a secretion signal peptide. Almost 76% of cellulases, hemicellulases and 88% of the accessory redox enzymes are targeted to secretion pathway, while only a very small amount remain inside the cell or represent membrane cell—bound macromolecules. Only 15.8% of the secreted enzymes in this review are predicted to possess a CBM and the majority of them comprise of auxiliary enzyme activities. The theoretical average molecular weight of secreted enzymes is 41.36 ± 15.9, varying between 10 and 97 kDa. The majority of secreted enzymes have molecular weight varying between 20 and 50 kDa, whereas β-xylosidases and β-glycosidases (GH3 family), and arabinofuranosidases (GH43 and GH51) appear to be high molecular weight proteins (Figure 5). The theoretical average isoelectric point of secretory enzymes is calculated 5.27 ± 0.8, at a range 4.34–7.9. *In vivo* expression and study of these enzymes would give different results, as the proteins are glycosylated, so size and pI value tend to moderate.

### Protein glycosylation

A total proportion of 92.8% of secreted proteins have either *N*- or *O*- putative glycosylation sites. These proteins are often glycosylated due to the existence of many Asn-Xaa-Ser/Thr sequons, which are known to be a prerequisite for *N*-glycosylation post-translational modifications. The molecules of many GHs and accessory enzymes have a modular structure consisting of a catalytic module, flexible peptide linker, and CBM. Flexible linker peptides, which are rich in Ser and Thr residues, are typically *O*-glycosylated (Gilkes et al., 1991). The *N*-glycosylation seems to be restricted to the catalytic modules, and it is usually absent in other parts of enzyme molecules. Various *N*-linked glycan structures have been found in different enzymes from *M. thermophila*, belonging to different enzyme classes and protein families (Gusakov et al., 2008). It has been noticed that glycosylation follows a heterogeneity pattern, meaning that in some molecules, the same Asn residue was modified with oligosaccharides having different structure, while not all of the potential glycosylation sites were found to be occupied. The most frequently met *N*-linked glycan was (Man)_3_(GlcNAc)_2_, a pentasaccharide which represents a well-known conserved core structure that forms mammalian-type high-mannose and hybrid/complex glycans in glycoproteins from different organisms (Dwek et al., 1993). Both types of glycosylation occur in 65% of secreted cellulases, 62.1% of secreted hemicellulases, while only *O*-glycosylation patterns appear in most of accessory enzymes. The presence of *N*-linked glycans is common for catalytic domain of the enzymes, while *O*-glycosylation usually occurs in linker region. Even though predicted to, non-secreted enzymes are not modified *in vivo* with glycans, since this procedure has been noticed as a post-translational modification in proteins targeted to the secretory pathway of the cell (Blom et al., 2004).

## Conclusions

Rapid depolymerization of lignocellulosic material is a distinguishing feature of thermophilic fungi, such as *M. thermophila*, which was isolated from soil and self-heating masses of composted vegetable matter (Domsch et al., 1993). However, the precise biochemical mechanisms and underlying genetics of this procedure are not completely understood. Systematic examination of the *M. thermophila* genome revealed a unique enzymatic system comprising of an unusual repertoire of auxiliary enzymes, especially those classified to AA9 family, and provided insights into its extraordinary capacity for protein secretion. The current review constitutes, to the best of our knowledge, the first genomic analysis of the lignocellulolytic system of *M. thermophila*. The genomic data, along with the observed enzymatic activity of several isolated and characterized enzymes suggest that this fungus possesses a complete set of enzymes, including 30 cellulases, 66 hemicellulases, and 35 proteins with auxiliary auxiliary enzymes, covering the most of the recognized CAZy families. From its cellulases to its oxido-reductases and multicopper enzymes, *M. thermophila* gene complement represents several avenues for further research and its diverse array of enzymatic capabilities will contribute to the study of lignocellulose degradation and the subsequent ethanol biofuel production.

### Conflict of interest statement

The Review Editor Ulrika Rova declares that, despite being affiliated to the same institution as authors Anthi Karnaouri, Io Antonopoulou, and Paul Christakopoulos, the review process was handled objectively and no conflict of interest exists. The authors declare that the research was conducted in the absence of any commercial or financial relationships that could be construed as a potential conflict of interest.
